# Diffuse Large B-Cell Lymphoma Presenting With Multiple Episodes of Acute Pancreatitis

**DOI:** 10.7759/cureus.82326

**Published:** 2025-04-15

**Authors:** Pavan J Patel, David Sokol

**Affiliations:** 1 Internal Medicine, Rutgers Robert Wood Johnson Medical School, New Brunswick, USA; 2 Hematology and Oncology, Penn Medicine Princeton Medical Center, Plainsboro, USA

**Keywords:** acute pancreatitis, diffuse large b cell lymphoma (dlbcl), lymphoma, malignant hematology, non-hodgkin lymphoma, recurrent acute pancreatitis

## Abstract

Gallstones and alcohol use are the most common causes of acute pancreatitis. When these etiologies are effectively ruled out, some less frequent causes include hypertriglyceridemia, medications, autoimmune conditions, and malignancy. Although rare, lymphoma infiltrating the pancreas can present with episodes of acute pancreatitis. In this report, we discuss a rare case of a patient who presented with multiple episodes of acute pancreatitis due to diffuse large B-cell lymphoma (DLBCL) infiltrating the pancreas. In the process, we emphasize the importance of maintaining a broad differential and performing the appropriate diagnostic evaluation when the immediate cause of acute pancreatitis is unclear.

## Introduction

Acute pancreatitis can have many different underlying etiologies, which are often readily apparent. Only about 10-15% of cases lack a clear explanation [1]. Among the typical causes, gallstones and alcohol use are the most common [1]. However, novel cases like the one described here provide a valuable learning opportunity when there is no clear etiology, at which point malignancy should be considered a possible underlying cause. Lymphoma with secondary involvement of the pancreas can occur in individuals with widespread nodal or extra-nodal disease [2]. Secondary involvement of the pancreas rarely presents as acute pancreatitis, with only a few cases noted in the literature [3-5]. In these prior instances, pancreatitis was the presenting symptom of the malignancy. Among these cases, one patient was observed to have multiple episodes of acute pancreatitis before the final diagnosis of lymphoma was established [5]. Here, we present the case of a 63-year-old male patient who was diagnosed with diffuse large B-cell lymphoma (DLBCL) following multiple episodes of acute pancreatitis due to pancreatic infiltration.

## Case presentation

**Figure 1:**
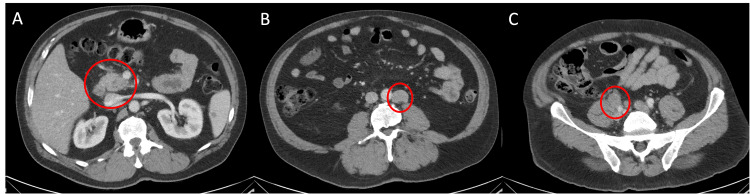
CT of abdomen and pelvis Axial view of the abdomen demonstrates haziness of the pancreatic head concerning for pancreatitis in image (A). Image (B) identifies the 2.7 x 2.0 cm enlarged left para-aortic lymph node. Image (C) identifies the enlarged right common iliac node, measuring 2.6 x 2.2 cm. CT: Computed tomography

A 63-year-old male patient presented to the emergency department due to a three-week history of periumbilical and epigastric abdominal pain that radiated to the back and worsened with eating. The pain was described as "burning", which the patient rated as an eight on a 10-point scale. He noted recent constipation but otherwise denied any further gastrointestinal (GI) or constitutional symptoms. His past medical history and family history were non-contributory. His social history was positive for alcohol use; however, none within the last week preceding presentation. He did not start any new medications prior to his symptom onset. Shortly prior to presenting, he had undergone biopsy of a right inguinal lymph node during a prior evaluation for his abdominal pain. He was afebrile, normotensive, and saturating 99% on room air. On physical exam, his abdomen was soft, non-tender, and without distention. The initial laboratory work-up was significant for an elevated lipase of 245 IU/L and a mildly elevated alanine aminotransferase (ALT) of 37 IU/L. His complete blood count (CBC), chemistries, and urine analysis were otherwise unremarkable. Computed tomography (CT) of the abdomen and pelvis was performed and revealed mild haziness around the pancreatic head concerning for acute pancreatitis without any pathology noted in the biliary tract. Retroperitoneal and right iliac adenopathy were also noted (Figure 1). He was admitted and treated for acute pancreatitis and was discharged on day two of his hospital stay.

Seven days later, he returned to the emergency department with a one-day history of epigastric pain that was identical to his prior symptoms. He denied any alcohol consumption in between hospital visits. His lipase was 425 IU/L, triglycerides 124 mg/dL, with a negative IgG4 subclass panel. He was treated with supportive care for a second episode of acute pancreatitis. During his second hospitalization, the pathology from his prior right inguinal lymph node biopsy demonstrated an enlarged lymph node with effacement by infiltrating large cells with high nuclear-to-cytoplasmic ratios and frequent mitotic figures. The immunohistochemistry demonstrated uniformly high expression of CD20, PAX-5, CD30, and CD45, without CD5, CD10, BCL2, BCL6, ALK-1, EBV, MUM-1, Cyclin D-1, CD138 and AE1/AE3. His Ki-67 was 70%, while the fluorescence in situ hybridisation (FISH) studies demonstrated BCL6 rearrangement without BCL2 or MYC rearrangements. In total, these findings were consistent with a new diagnosis of DLBCL.

**Figure 2:**
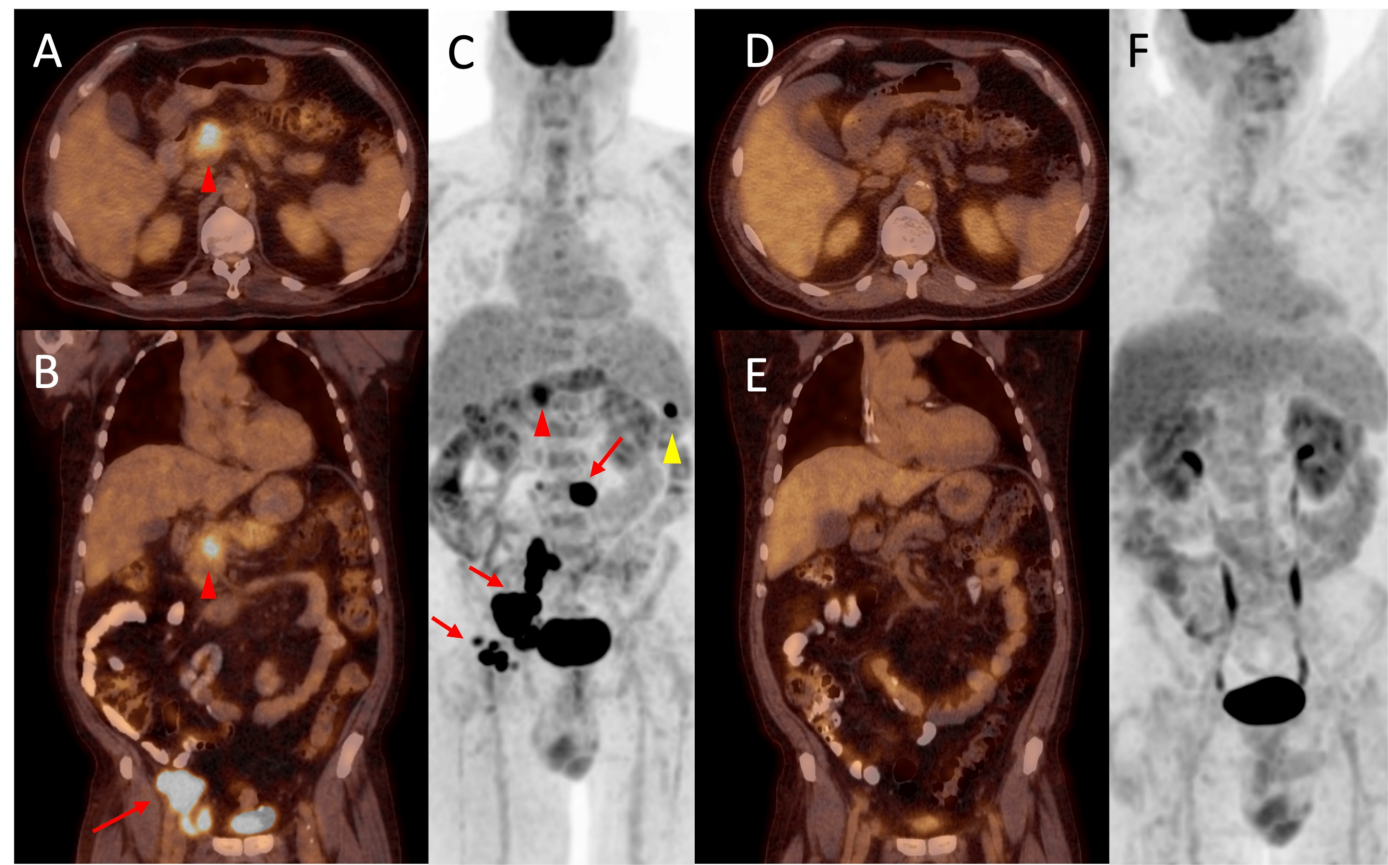
Fused PET/CT images with axial view of the chest and coronal view of the chest/abdomen/pelvis, along with coronal MIP images, before and after chemotherapy Images (A), (B), and (C) demonstrate hypermetabolic lesions in the uncinate process of the pancreas (red arrowhead), spleen (yellow arrowhead), and multiple lymph nodes, including the left para-aortic, right iliac, and right inguinal nodes (red arrows). Following three cycles of chemotherapy, images (D), (E), and (F) demonstrate resolution of the previously noted hypermetabolic activity. PET/CT: Positron emission tomography/computed tomography; MIP: Maximum intensity projection

There was now concern that his unexplained episodes of recurrent pancreatitis were related to this new diagnosis. Positron emission tomography (PET) was performed and demonstrated hypermetabolic adenopathy and a lesion within the uncinate process of the pancreas concerning for lymphomatous infiltration (Figure 2). With the PET findings confirming pancreatic involvement, he was considered stage IV as per the Ann Arbor staging system at diagnosis. He received supportive care and was discharged after improvement of his pain with oncology follow-up as an outpatient. He received six cycles of R-CHOP (rituximab, cyclophosphamide, doxorubicin, vincristine, prednisone) chemotherapy, with resolution of his symptoms and complete radiographic resolution on positron emission tomography/computed tomography (PET/CT) following cycle three (Figure 2). More than one year after his initial diagnosis, the patient remained without further episodes of pancreatitis and did not demonstrate any evidence of disease recurrence. 

## Discussion

The presentation for a patient with non-Hodgkin lymphoma may include hematologic abnormalities, adenopathy, and, less frequently, constitutional symptoms. However, in this case, the malignancy presented with multiple episodes of acute pancreatitis in the context of lymphadenopathy without any significant hematologic abnormalities. Extra-nodal DLBCL involving the pancreas, leading to episodes of acute pancreatitis, is a rare clinical phenomenon with few cases documented in the existing literature [3-5]. The patient discussed here contributes to this relatively small number of reported cases. Additionally, it is noteworthy that he experienced multiple episodes of acute pancreatitis, similar to a specific reported case [5]. Another unique feature of this patient’s case is that his immunohistochemistry showed high expression of CD30. Although this marker is more commonly discussed in the context of Hodgkin lymphoma, one study involving 903 patients with DLBCL treated with R-CHOP found that CD30 positivity was associated with improved progression-free survival and overall survival at five years [6]. In the case presented here, the patient’s disease responded favorably to R-CHOP, with complete radiographic resolution after three cycles. At over one year of follow-up, he had not experienced any further episodes of acute pancreatitis and showed no evidence of lymphoma recurrence.

Beyond the novelty of the clinical case, this patient’s presentation offers a valuable opportunity to enhance our understanding of the diagnosis of acute pancreatitis. By meeting all three of the Atlanta criteria for acute pancreatitis (characteristic pain, imaging findings, and lipase elevation), the patient’s clinical profile appeared typical for his condition [7]. However, the primary challenge with the initial diagnosis was the difficulty in identifying a clear inciting cause. His initial laboratory findings did not show significant changes in liver function, making an obstructive cause unlikely; this was later confirmed by CT. During the initial evaluation during the first hospital stay, alcohol use seemed a potential cause, as the patient reported recent alcohol consumption. However, in subsequent discussions with the patient, he clarified the initial clinical history and revealed that his symptoms began prior to his alcohol use. Notably, his second episode of acute pancreatitis occurred without any alcohol consumption between hospital stays. At this stage, the two most common etiologies of acute pancreatitis - gallstones and alcohol use - had been effectively ruled out. Other less common causes, including hypertriglyceridemia, medication-induced factors, and autoimmune-related issues, were also assessed. Despite these evaluations, the etiology of his multiple episodes of acute pancreatitis remained uncertain until his lymph node biopsy results were available. Nevertheless, even with the pathologic findings of DLBCL, this patient’s case had not been fully reconciled. The most common sites outside of lymph nodes for DLBCL involvement are the spleen, thymus, and Waldeyer’s ring [8]. As mentioned earlier, pancreatic involvement is exceedingly rare; therefore, his recurrent episodes of acute pancreatitis could not be linked to his lymphoma until a PET scan revealed findings consistent with pancreatic infiltration by the lymphoma.

## Conclusions

The diagnostic clues for this patient’s true cause of pancreatitis were present during his initial evaluation. He informed the medical team about the lymph node biopsy, and his initial CT scan revealed adenopathy. At this stage, it would have been appropriate to give more consideration to malignancy as a cause for his symptoms, but the history of alcohol use in the patient with new acute pancreatitis seemed typical without requiring further workup. In his case, a rare manifestation of extra-nodal lymphoma was the underlying cause of his recurring symptoms. His case serves as a strong reminder to remain vigilant against diagnostic anchoring. Despite being a rare entity, extra-nodal lymphoma should be considered on the differential for causes of acute pancreatitis, particularly in the absence of another clear etiology.
